# Advances in diagnosis of diseases causing diarrhea in newborn calves

**DOI:** 10.1007/s11259-025-10855-0

**Published:** 2025-08-28

**Authors:** Doaa Sedky, Alaa A. Ghazy, Hala A. A. Abou-Zeina

**Affiliations:** Department of Parasitology and Animal Diseases, National Research Centre, Veterinary Research Institute, Giza, Egypt

**Keywords:** Diarrhea, Newborn calves, Diagnosis, ELISA, Immunochromatographic tests, PCR, LAMP

## Abstract

Diarrhea in newborn calves is a serious global health problem. It poses challenges for animal industry, veterinarians and researchers due to the rapid onset of dehydration. Mixed infections make treatment complicated, and many young calves suffer high rates of illness and death from this condition. Numerous enteropathogens are associated with diarrhea in newborn calves, encompassing viruses, bacteria, parasites, and protozoa. Their occurrence differs by region, yet the most prevalent infections include *E. coli*, *Salmonella* species, *Clostridium perfringens*,* Clostridium difficile*,* Rotavirus*,* Coronavirus*,* Cryptosporidium*,* Toxocara*,* Giardia* and *Eimeria*. This review outlines the diagnostic techniques for diseases that lead to diarrhea in newborn calves. Diagnosis is based on clinical manifestations; however, the laboratory identification of etiological items is the only valid way for detecting the illness’s aetiology and initiating treatment protocols. Classic methods such as bacterial culturing, fecal flotation, direct microscopy, and virus isolation help us understand pathogens better. Immunological assays like ELISA and immunochromatography are fast, accurate, affordable, and useful for on-farm detection. They help identify specific antigens or antibodies efficiently. Molecular methods including PCR (standard, multiplex, real time and digital), LAMP assays, DNA microarrays and whole-genome sequencing allow highly accurate and sensitive detection. They can identify pathogens effectively, even at very low levels. Nanotechnology-based assays introduce a novel level of sensitivity and specificity, often yielding quick results with minimal sample volumes. In conclusion, accurate and rapid diagnosis using advanced techniques is critical for managing and preventing diseases that lead to diarrhea in newborn calves.

## Background

Diarrhea in newborn calves aged 1-month-old or younger is one of the most prevalent and economically disastrous health issues encountered in the livestock sector worldwide (Meganck et al. 2014; Fouad et al. 2024). The mortality rate of calves experiencing diarrhea in the early weeks post-birth represents around 57% of overall mortality, notably during the third week after birth (Mohamed et al. 2017; Chen et al. 2023). Key pathogens linked to diarrhea in calves include *bovine Rotavirus* group A (BRV-A), *bovine Coronavirus* (BCoV), *bovine Norovirus* (BNoV), *Nebovirus*, *Salmonella*, *E. coli* K99, and *Cryptosporidium parvum* (*C. parvum*) (Cho et al. 2013; Sobhy et al. 2020; Garro et al. 2021). Among these, Cho et al. (2013) found that *C. parvum* and *BRV-A* were the most common, with high detection rates of 33.7% and 27.1%, and significant odds ratios of 173 and 79.9, respectively. BNoV and Nebovirus were also important, with Nebovirus showing a high odds ratio of 16.7, indicating a significant impact on calf diarrhea. Mixed infections are commonly reported and often lead to more severe disease in newborn calves (Pansri et al. 2022). Affected calves suffer from dehydration, electrolyte imbalances, and metabolic acidosis, which, if left untreated, can lead to death (Caffarena et al. 2021). Accurate and rapid diagnostic tests are of tremendous value leading to better treatment and prevention outcomes (Altuğ et al. 2013; Chen et al. 2023). The most common diagnostic techniques for enteric infections in calves include culture, biochemical recognition, and microscopic inspection, all of which are arduous, time-consuming, and frequently lacked both specificity and sensitivity. Further, immunologic diagnostics utilising antibody or antigen recognition may provide great sensitivity and specificity. Examples of these assays are Enzyme Linked Immunosorbent Assay (ELISA), direct ELISA, indirect ELISA, sandwich ELISA, Competitive ELISA and Immunomagnetic Separation (Hamedian et al. 2022; Uslu et al. 2023). Immunochromatographic assays are widely used in veterinary medicine. They are valued for their accuracy, low cost, quick results, and simplicity for on-farm use (Icen et al. 2013). These tests are one of the new technologies that have gained a lot of attention for their ability to provide quick diagnoses (Altuğ et al. 2013; Icen et al. 2013; Keles et al. 2022). Advancements in molecular diagnostics built on nucleic acid recognition methods, including conventional polymerase chain reaction (PCR), real-time PCR, multiplex PCR, isothermal amplification tests, and genome sequencing. Recently, these methods have become more common for detecting, characterizing, and identifying diseases that cause diarrhea in newborn calves (Algammal et al. 2020; Ji et al. 2023). In a study performed by Wei et al. (2021), the PCR results showed that the percentages of diarrheic newborn calves samples that were positive for *C. parvum*, *Bovine Rotavirus* A, *Bovine Coronavirus*, and *Giardia lamblia* were 44.93, 36.23, 17.39, and 13.04%, respectively. Meanwhile, the rapid kit immnochromatographic assay results showed that the prevalence of *C. parvum*, bovine *Rotavirus* A, bovine *Coronavirus*, and *Giardia lamblia* was 52.17, 31.88, 28.98, and 18.84, respectively. Isothermal amplifying tests like Loop-Mediated Isothermal Amplification (LAMP) and recombinase polymerase amplification could help create diagnostic tools for livestock diseases and are suitable for field use because they operate at the same temperature (Meng et al. 2024). Nanotechnology-based assays bring a novel level of sensitivity and specificity, delivering quick results with small sample volumes (Razmi et al. 2020). This review aims to discuss the diagnostic methods for diseases that cause diarrhea in newborn calves.

## Diagnosis of diseases causing diarrhea in newborn calves

Diarrhea can develop rapidly, so it’s important to diagnose it right away. This helps veterinarians find out the cause and take the right actions to treat it effectively.

### Clinical history

A clinical history can help diagnose, prevent, and treat diarrhea in newborns calves. The history includes data on vaccination, age, colostrum consumption, and the diarrhea’s duration and its severity, along with the number of sick or dead animals in the herd (Muktar et al. 2015). In addition to assessment of housing, management, nutrition, sanitation, and prevention strategies is also important. On-farm standard operating procedures regarding treatment protocols are important to obtain and review, especially when approaching outbreaks of diarrhea. It is also important to ascertain whether there have been any recent dietary or husbandry changes (weaning), transportation, on-farm treatments, or addition of new animals. There is a considerable association between age and the detection of specific infections (Cruvinel et al. 2020; Keles et al. 2022).

Table 1 shows how the types of pathogens causing diarrhea vary with age, especially in young calves. F5 (K99) *E. coli* is a common cause of diarrhea in newborns under 7 days old. *Cryptosporidium* appears in feces only after 3 days, while *Coccidia* are found after 15 to 21 days owing to their development stages. Blood in the feces of calves younger than 30 days is often linked to *Salmonella*, *Coronavirus*, and less frequently to *Cryptosporidium* and *Clostridium* (Berber et al. 2021a; Diaz et al. 2021; Hamedian et al. 2022).

**Table 1:** Age-associated pathogen distribution

Agent	Typical Age at Infection	Refrence
*Enterotoxigenic E. coli*	0–7 days	(Coura et al. 2015; Keles et al. 2022)
*enterohemorrhagic E. coli*	2 days–4 weeks	(Coura et al. 2015)
*Rotavirus*	5–12 days (up to16 weeks in small ruminants differ depending on serogroup)	(Hamedian et al. 2022; Keles et al. 2022 )
*Coronavirus*	5 days − 1 month	(Cruvinel et al. 2020; Keles et al. 2022 )
*Cryptosporidium*	1–4 weeks	(Diaz et al. 2021; Keles et al. 2022 )
*Clostridium perfringens* type C	3–14 days	( Jessop et al. 2024 )
*Clostridium perfringens* type A	2–4 weeks old	( Daly and Rotert 2007 )
*Salmonella*	2–6 weeks of age	(El Seedy et al. 2016)
*Giardia*	2 weeks–2 monthes	(Cruvinel et al. 2020)
*Toxocara vitulorum*	3 weeks – 2 months	(Celik et al. 2022)
*Coccidia*	2 weeks- 3 weeks	(Abdou et al. 2021)

### Physical examination

Diarrhea must be carefully assessed, with the duration and severity of diarrhea as primary considerations during the physical examination, as its color, odor, and amount provide diagnostic insights. Observing straining, blood, or mucus in stools can indicate serious gastrointestinal problems, helping doctors evaluate the illness severity and determine treatment (Lotfy et al. 2020; Ekinci et al. 2022; Elsadek et al. 2022). *E. coli* K99 + causes a watery diarrhea while the diarrhea caused by *Salmonella* infection is characterized by watery and mucoid diarrhea with the presence of fibrin and blood (Muktar et al. 2015). Degree of dehydration and clinical signs in newborn calves are shown in Table 2 according to Smith (2005); signs include sticky or dried mucous membranes, reduced skin elasticity, and sunken eyes. In young animals with enteritis, signs like discomfort, back arching, hind limb stomping, or lying down with legs extended may appear. Signs of metabolic acidosis may be observed as shown in Table 3; the degree of acidosis based on posture, behavior and palpebral reflex of the calf (Meganck et al. 2014). Variations in behaviour, posture and palpebral reflex are more closely correlated with elevations of serum D-lactate concentrations than with decreases in base excess (BE) (Lorenz 2004; Meganck et al. 2014). In sever cases, animals are reluctant to stand, struggle with suckling, lying down and show signs of mental problems, like being less alert or very tired (Constable et al. 1998; Constable 2004). Rectal temperature is important for assessing the severity of illness in newborns calves with enteritis, indicating conditions like fever, normal, or hypothermic levels. Hypothermia signals potential struggles, often linked to low blood sugar, and is concerning, especially with metabolic acidosis. To diagnose endotoxemia or septicaemia, check the colour and capillary refill rate of mucosal membranes and look for dilated blood vessels in the sclera. Septicaemic calves may show hypopyon in the eye, indicating severe infection. Early detection of clinical symptoms is crucial for prompt intervention **(**Smith et al. 2020; Ekinci et al. 2024).

**Table 2:** Degree of dehydration and clinical signs (Smith 2005)

Degree of Dehydration	Estimated Fluid Loss (%)	Clinical Signs
Mild	6–8%	slight eyeball recession, skin tent slightly prolonged (2–4 s), mucous membranes moist
Moderate	8–10%	eyes obviously sunken, skin tent obviously prolonged (4–8 s), mucous membranes tacky, dull or lethargic calf
Severe	10–12%	eyes severely sunken into orbits, skin remains tented indefinitely, mucous membranes dry; shock-like symptoms
Very Severe	> 12%	Recumbency, loss of consciousness, shock, or near death

**Table 3:** Clinical Estimation of metabolic acidosis in diarrheic calves ( Meganck et al. 2014 )

Category	Clinical Sign	Estimated base excess (mmol/L)
Posture	Standing up by itself	–2.2
	Standing after encouragement	0.0
	Standing steadily after lifting	+ 2.8
	Standing unsteadily; corrects if forced	–11.7
	Standing unsteadily; unable to correct if forced	–20.6
	Unable to stand – sternal recumbency	–20.9
	Unable to stand – lateral recumbency	–25.4
Behavior	Bright and alert; reacts to acoustic & optical stimuli	+ 2.5
	Adequate reaction to stimuli	+ 1.8
	Delayed reaction to stimuli	–14.6
	Reacts only to painful stimuli	–21.0
	No reaction even to painful stimuli	–25.4
Palpebral Reflex	Eyelids close immediately and fully	+ 0.7
	Eyelids close immediately but not fully	–7.6
	Eyelids close with delay and not fully	–19.9
	Eyelids do not close at all	–24.3

### Laboratory diagnosis

Several microorganisms or factors can cause diarrhea, hence lab analysis is required for an appropriate diagnosis. There’s no single test that can identify a pathogen perfectly; each technique has limits, as shown in Table 4.

**Table 4:** Diagnostic assays used in the detection of enteric pathogens in newborn calves

Method	Samples	Advantages	Disadvantages	References
Fecal bacterial culture	-Rectal swabs, feces, and intestinal tissues at necropsy.	-Antibiotic susceptibility testing (AST) to determine the most effective treatment. - less expensive -Vaccine development	- Inadequate specificity - Slow return time - Infectious bacteria must exist. - Time-consuming	( Muktar et al. 2015 )
Fecal flotation and direct microscopy	-Rectal swabs, feces, and intestinal tissues at necropsy.	-Typically utilised for parasites, eggs, and oocytes. - Fast detection. -Low budget.	- Low sensitively - Requires the optimum quantity of oocysts. - Subjectivity interpretation of results.	(Abu El Ezz et al. 2020)
Virus Identification (Immuno-Fluorescence Assays and EM)	-Largely tissue samples. - EM: Rectal swabs, and faeces can be utilised with appropriate processing techniques.	- Validation of existence of infectious virus within clinical samples. - The availability of an isolated virus for further characterisation or vaccine manufacturing. - Immuno-electron microscope has greater sensitivity than direct TEM.	-Direct detection relies on accurate sample collecting and analysing to aid diagnosis. -It needs a high viral concentration. -Time consuming and pricey. -The sensitivity might range from low to moderate, depending on the sample and test. -The specificity is modest at the species level. -Not appropriate for cytotoxic samples.	( Kassem et al. 2017 )
ELISA, Immuno-chromatographic assays	-Serum, plasma, and milk (colostrum). Feces for antigen test.	-High specificity and sensitivity. -Fast and ideal for large numbers of samples (herd testing). -Commercial kits, some of which include lateral flow testing for faster results. -Allows some degree of quantifying. -Could be used to identify antibodies and antigens.	-Be cautious when interpreting results because antibodies may not be indicative of a current infection. -Inadequate sensitivity to analysis -In some cases, it is prohibitively expensive. -Specificity issues brought on by background signal or non-specific binding - In certain situations, PCR confirmation may be necessary. -The specificity and sensitivity are lower than those of molecular testing.	(Hamedian et al. 2022)
PCR, Real-time PCR- Multiplex –PCR- qPCR	-Rectal swabs, feces, and intestinal tissues at necropsy.	-Excellent specificity and sensitivity (gold-standard assay). -Quick outcomes and excellent accuracy with verified systems. -qPCR enables real-time analysis and some pathogen load quantification. -Multiplexes and panels aid in the diagnosis of disease syndromes. - Most commonly applied assay. - Presence of an antigen does not always correspond with disease, thus quantification is beneficial.	-Experienced staff is needed. -The possibility of contamination when processing the sample. -Untrue negative results brought on by recombination or genetic mutation.	( Golaviya et al. 2024 )
Isothermal Amplification Assays (LAMP, RP)	-Rectal swabs, feces, and intestinal tissues at necropsy.	-Excellent specificity and sensitivity. -Quick and precise findings. -Equipped with quick readout devices (lateral flow strips, for example). -Less demand for lab apparatus. -Multiplexing is an option.	-Techniques are still being developed to offer a comprehensive assay that can be extensively used and incorporated into practice.	( Karakavuk et al. 2024; Liu et al. 2022 )
Microarray and Sequencing	-Sample with high quality nuclear material.	-Extremely high specificity and sensitivity. -The ability to describe variations (outbreak condition). -Can identify numerous pathogens immediately (multiplex) with less constraints.	-Not extensively utilised in diagnostics right now. -Slower along with more costly than other tests. -Requires high-quality nucleic acids.	( Wang et al. 2010 )

*Abbreviations* *EM* Electron microscopy, *PCR* Polymerase chain reaction, *qPCR* quantitative Polymerase Chain Reaction, *ELISA* Enzyme-linked immunosorbent assay, *LAMP* Loop-mediated isothermal amplification, *RPA* Recombinase Polymerase Amplification

#### Collection of samples

The handling and transport of specimens to a diagnostic laboratory are essential for accurate results. For fecal samples from calves with diarrhea, fresh fecal samples should be collected using rectal swabs to minimize contamination (Su et al. 2022). Proper storage in icebox at 4–8 °C, particularly in transport media, helps keep the sample intact and prevents decay or bacterial growth. Anaerobic bacteria, like *Clostridium perfringens*, require oxygen-free environments to prevent degradation. Keeping all samples cold slows down microbial activity, preserving their state for laboratory testing (Ismael et al. 2019). Specimens from fresh sacrificed, dead or euthanised calves are beneficial for diagnosis during epidemics. It’s important to collect fresh and formalin-fixed gastrointestinal tissues, lymph nodes, and liver samples (Hamouda et al. 2014). Serum samples are also collected for immunological based assays. These samples can help with immunological evaluations. Blood or milk samples show exposure to infections through antibodies, which is valuable for assessing health in both individual dairy animals and the entire herd (Ata et al. 2020).

#### Conventional diagnostic assays

##### Bacterial culturing

Bacterial culturing is a gold standard lab technique used to isolate and identify bacterial pathogens from fecal and intestinal samples (Ata et al. 2020). Various agar plates such as blood agar, MacConkey agar and Hektoen enteric (HE) plates are essential for this process. Media like brain heart infusion (BHI) broth facilitate general bacterial growth, whereas selective broths like tetrathionate are intended to isolate specific pathogens such as *Salmonella*. Blood agar is extensively used because it encourages the growth of a wide variety of bacteria. MacConkey agar is selective for Gram-negative bacteria and aids in differentiating lactose fermenters. Sorbitol-MacConkey agar is specifically used to distinguish non-pathogenic *E. coli* versus the pathogenic *E. coli* O157:H7, that can’t ferment sorbitol. For *C. perfringens*, thioglycolate broth is applied in anaerobic conditions at 36 °C for two days (Quinn et al. 2002). The colonies that develop on the medium will be analyzed for colony morphology, physical characteristics, microscopic features, and biochemical and serological detection. A test kit for biochemical identification, API 20E and the Biolog automated microbial identification systems are two examples of commercial biochemical tests that have a sensitivity of 79% (Peele et al. 1997). The causes of diarrhea in calves were studied by Fouad et al. (2024) using bacterial cultures. They analyzed 364 fecal samples and identified 356 bacteria, with the most common being *E. coli* (35. 99%), *Salmonella enterica* (24. 72%), *S.* Typhimurium (13. 73%), *S.* Dublin (10. 99%), *Citrobacter diversus* (9. 61%), *Klebsiella pneumoniae* (6. 59%), *Proteus vulgaris* (5. 49%), *Proteus penneri* (5. 22%), and *Staphylococcus aureus* (10. 16%). A study carried by Ismael et al. (2019) looked at the presence of *C. difficile* and its toxins in different animals. They collected 249 fecal samples from cows, buffaloes, sheep, goats, horses, dogs, and cats, both healthy and sick. *C. difficile* was found in both healthy and sick animals, but not in healthy goats, buffaloes or cats. One hundred and twenty fecal samples from calves under three months old with diarrhea during Egypt’s dry and rainy seasons were studied by Elhady et al. (2020). They found that 71 samples (59. 5%) tested positive for *E. coli*, and 36 samples (30%) for *Salmonella*, with 26 samples (21. 66%) showing both infections. *Salmonella* can be cultured using specific plates like *Salmonella*-*Shigella* and bismuth sulfite agar. The International Organisation of Standards (ISO) has set guidelines for *Salmonella* screening (Mooijman et al. 2019). Pre-enriching samples with buffered peptone water, then using Rappaport Vassialidis soy broth and Muller Kauffmann tetrathionate-novobiocin for selective enrichment then enriched sample is then streaked on other media like Xylose Lysine Deoxycholate (XLD) and Hoektoen; the ISO method has a sensitivity of about 87%, while the PCR method has a sensitivity of 83.6% and a specificity of 97.4% (Mainar-Jaime et al. 2013). The presence of *Salmonella* and *E.coli* in diarrheic calves was studied by El-Seedy et al. (2016). They took faecal samples from 127 calves up to three months. They found 119 bacterial isolates, with *Salmonella* detected in 18.1% and *E. coli* in 75.6% of the samples. The most common *Salmonella* types were *S.* Enteritidis and *S.* Typhimurium, while *E. coli* revealed ten O-serogroups, with O26 and O103 being the most frequent. Disadvantages of traditional bacteria culture methods for testing bacteria are slow and time-consuming, which can delay treatment decisions in addition to inadequate specificity (Muktar et al. 2015). New technologies like PCR and other molecular diagnostics provide quicker results (Algammal et al. 2020; Keles et al. 2022).

##### Fecal flotation and direct microscopy

Fecal flotation, fecal smears and direct microscopy are common methods for detecting intestinal eggs or oocysts, but they have some limitations. They may not be very sensitive and require enough oocysts for reliable results. Multiple tests might be needed, especially if oocysts are shed at different times. Results can also vary based on the observer’s skill and staining methods used. These issues show that trained staff and better procedures are important for more accurate diagnoses (Dryden et al. 2005). To identify *Cryptosporidium parvum*, labs often look for oocysts in feces or stained intestinal tissue, and multiple samples from the same animal may be necessary since some animals only shed oocysts periodically (Omoruyi et al. 2014). Traditional acid-fast staining techniques like Kinyoun and Ziehl-Neelsen, stain oocysts bright red, while other materials turn green or blue. Newer staining methods, like auramine-rhodamine, only target oocysts and are very sensitive, though more expensive (Ghazy et al. 2015). Studies have shown that PCR testing is more effective than microscopy in detecting these parasites. Standard methods like fecal flotation, fecal smears and direct microscopy face challenges due to sensitivity limits and subjective interpretation, which can impact diagnosis. One hundred and seventy-two faecal samples from diarrhoeic pre-weaned bovine calves in Giza and Sharkia, Egypt, were examined by Abu El Ezz et al. (2020) for *Cryptosporidium* oocysts using modified Ziehl-Neelsen microscopy. They found 45. 9% (79) of the samples were positive, while molecular testing with nested PCR confirmed the *C. parvum* isolates with 100% sensitivity. Additionally feces from 32 pregnant ewes on the first three days after delivery were tesed by Santín et al. (2007), as well as from each of their lambs on days seven, fourteen, and twenty-one after birth. They used microscopy and PCR/gene sequence analysis to find *Cryptosporidium* oocysts and *Giardia* cysts, with PCR being more sensitive than microscopy and the detection rates for *Cryptosporidium* were 25% in ewes and 77.4% in lambs.

##### Virus isolation

Virus isolation is considered as the gold standard method for detecting viral pathogens because it involves growing the virus in a controlled setting, which confirms its presence (Ates and Yesilbag 2023). Different cell cultures are used depending on the virus. For example, MDBK cells, MA104 cells, TF, and PK-15 cells are often used to isolate *Rotavirus* and *Coronaviruses* from animal feces. The effectiveness of viral isolation can vary based on how susceptible each cell line is to specific viruses (Makwana et al. 2020). Fifty fecal samples from diarheic calves under three months old were tested by Kassem et al. (2017) and successfully isolated Rotavirus from five samples (10%) using MDBK cell inoculations. This process showed specific cell changes, such as rounding and increased granularity, leading to grape-like clusters in 72–96 h. Previous studies found that adding trypsin to MDBK cell cultures can increase Rotavirus growth by 100 times (Garaicoechea et al. 2006). Viral isolation has benefits like confirming the presence of a virus in samples, providing purified virus for research, and helping create diagnostic kits and vaccines. However, there are limitations that include sensitivity issues, varying cell capabilities, dependence on proper sample collection and storage, and inapplicability for cytotoxic samples (Golaviya et al. 2024). Embryonic eggs and lab animals are also used to isolate and grow *Coronavirus*es and Rotaviruses (Guy 2015).

##### Electron microscopy (EM)

Electron microscopy (EM) is an important method for quickly diagnosing viruses in animals. Its primary benefit is its capacity to directly view viral morphology, which is especially beneficial for enteric viruses such as Rotavirus and *Coronavirus* (Akilesh et al. 2021). These viruses are frequently difficult to cultivate in cell cultures, yet EM can distinguish them by their unique shapes and sizes (Athanassious et al. 1994; Soltan et al. 2016). A study in Iran by Davoudi et al. (2014) assessed the presence of bovine *Coronavirus* in fecal samples from calves with diarrhea, finding it in 15% of cases, with peaks in winter (28%) and low in summer (4%). The study highlighted EM as the best diagnostic technique, allowing direct observation of virus shapes. However, EM has notable limitations. It requires a high concentration of virus particles, ranging from 10^4^ to 10^6^ per milliliter, which makes it a low-sensitivity method. Additionally, it cannot analyze multiple samples at once, limiting its use in high-throughput testing settings. The high cost of electron microscopes and the need for specialized lab staff also restrict its use as a standard diagnostic tool. Despite these issues, EM is beneficial in certain cases where quick visualization of hard-to-culture viruses is needed (Catroxo et al. 2010).Transmission electron microscopy (TEM) is a key method for viewing virus particles in fecal or intestinal samples through positive and negative staining methods (Gallagher et al. 2019). This technique is vital for quick viral diagnosis in animals and is seen as the best method for diagnosing enteric viruses (Martella et al. 2020; Berber et al. 2021b). However, TEM has some drawbacks; it needs a high number of virus particles, can’t handle multiple samples at once, and is expensive. Still, TEM plays a crucial role in visualizing viruses that are hard to culture. Fifty nine fecal and 10 intestinal samples from a diarrhea outbreak in capybaras in Piracicaba, Brazil were analysed Using TEM (Catroxo et al. 2010), the study found many *Coronavirus*-like particles with pleomorphic, round, or elongated shapes and a Corona of radial projections (80–140 nm in diameter).

#### Immunological assays

Immunodetection is now a popular approach for detecting intestinal infections due to its sensitivity and specificity. It uses immunological assays that rely on antibodies to detect bacteria, viruses, spores, and toxins (Ramos et al. 2020). These methods depend on the interaction between antigens and antibodies, utilizing both polyclonal and monoclonal antibodies. While immunological detection may not be as specific or sensitive as nucleic acid-based methods, it is faster and more effective, capable of identifying both harmful organisms and their toxins that might not be in the organism’s genetic material (Uslu et al. 2023).

##### Enzyme-linked immunosorbent assay (ELISA)

An enzymatic immunosorbent assay (ELISA) is a test that uses antibodies and enzymes to detect specific substances. There are several types of ELISA, including direct, indirect, sandwich, competitive, and immunomagnetic separation (Lee et al. 2018; Pang et al. 2018; Tabatabaei and Ahmed 2022). Additionally, ELISA used to check 300 fecal samples from diarrheic neonatal calves for pathogens (Uslu et al. 2023). They found *Cryptosporidium* oocysts in 36.3% of samples, Rotavirus in 19%, *E. coli in* 5.6%, and *Coronavirus* in 2% of samples. Mixed infections were also noted. A study by Manuja et al. (2010) assessed different tests for group A Rotavirus in buffalo calves, finding that ELISA, RNA-PAGE, and RT-PCR tests showed different levels of effectiveness when checked against viral isolation. ELISA had a sensitivity of 100%, while RNA-PAGE and RT-PCR had 66.67% and 71.43%, respectively. The specificity was 97% for RNA-PAGE, and both RT-PCR and ELISA had a specificity of 100%. ELISA is quick, easy and sensitive, suitable for routine lab testing and field research. Five hundred and fifty nine lambs under 30 days old from 30 sheep folds in northern Algeria were tested for pathogens using antigen ELISA (Dahmani et al. 2020). The results showed high rates of infection *Cryptosporidium parvum* was found in 58.81% of the lambs, *E. coli* K99 in 27.72%, Rotavirus in 12.88%, and *Coronavirus* in 3.57%. *Cryptosporidium parvum* was the most common, especially in lambs during the second and third weeks of life, while other pathogens were more prevalent in lambs younger than 7 days old. Moreover a dipstick ELISA kit was used to detect *E. coli* K99 in diarrheic calves (Muktar et al. 2015), finding a sensitivity of 71. 4% and a specificity of 100% compared to PCR. They noted that serology could help in diagnosing *Salmonella*, mainly for assessing herd levels. The detection limit of Rotavirus and *Coronavirus* in calf faeces and the sensitivity of the antigen-capture ELISA are lower than RT qPCR, mainly in subclinical infections, and neutralizing anti bodies derived either from colostrum or active immune responses may interfere with viral detection by antigen-capture ELISA (Izzo et al. 2012). Commercial ELISA kits are available for various pathogens (Izzo et al. 2012; Khurana and Chaudhary 2018; Pang et al. 2018), but they exhibit lower sensitivity than culture methods, emphasizing the necessity to obtain samples from severely ill animals (Mirhashemi et al. 2015). These kits may be costly and cannot distinguish between species or subtypes. Antibody responses can take time to develop, with IgA and IgM indicating recent exposure, while IgG shows past exposure. Measuring antibody levels helps understand exposure and informs treatment and vaccination approaches (Woolums 2023).

##### Immunochromatographic assays (ICG)

Rapid identification of intestinal pathogens can help in managing treatment. While methods like electron microscopy, viral culture, ELISA, and staining techniques can identify pathogens such as Rotavirus, *Coronavirus*, *E. coli*, and *Cryptosporidium*, they are complicated, require special equipment, and take a lot of time. A new diagnostic method using lateral immunochromatography has also been created for detecting Rotavirus, *Coronavirus*, *E. coli* f5 (K99), and *Cryptosporidium* (Altuğ et al. 2013; Hamedian et al. 2022; Keles et al. 2022). This method has the benefit of not demanding special equipment or experience, making it ideal for small labs and field studies. In veterinary medicine, several diagnostic platforms are used notably the tube technique, microtiter plate method, and membrane-bound approach. The microtiter plate method is widely used in diagnostic labs because it allows for testing many samples at once. In contrast, the membrane-bound method, using a lateral flow approach is often used in point-of-care settings (Lichtmannsperger et al. 2022). Lateral flow assays, like strip tests, SNAP tests, and rapid kits, are popular in clinics or near patients due to their ease of use, quick results, and portability. Faecal samples from 192 diarrheic and 14 healthy calves, aged 2 to 40 days, were tested by Icen et al. (2013) for bacterial, viral and parasite infections. They found that four rapid assays detected enteropathogens in 92.7% of the diarrhea cases. The individual prevalences were *Rotavirus* (25%), *Cryptosporidium parvum* (21.8%), *E. coli* K99 (9.4%) and *Coronavirus* (2.1%). Coinfections were common, with combinations like *Rotavirus* + *Cryptosporidium* (15.6%), *Rotavirus* + *E. coli* K99 (7.3%), and *Cryptosporidium* + *E. coli K99* (5.2%). Some calves had infections from three or even all four pathogens. These findings show that diarrhea in calves is complex and emphasize the need for targeted treatments and preventive measures **(**Keles et al. 2022; Sayar and Keles 2024). Recent tests have been developed to quickly identify *C. parvum* and other key pathogens in the feces of diarrheic calves. These immunochromatographic assays can detect *C. parvum* antigens in a few minutes without needing to concentrate the samples, making them useful for fieldwork. Three commercial kits were evaluated by Papini et al. (2018) for detecting *C. parvum* in newborn calves, measuring factors like sensitivity and specificity. Results showed the FASTest^®^ CRYPTO-GIARDIA Strip had the highest infection rate at 65.15%, followed closely by the FASTest^®^ CRYPTO strip (63.64%) and TETRASTRIPS^®^ (56.06%). Both FASTest CRYPTO strips and the FASTest^®^ CRYPTO-GIARDIA Strip had perfect sensitivity (100%) compared with TETRASTRIPS (90.24%), and TETRASTRIPS^®^ had the best specificity (100%) compared with FASTest CRYPTO strips (96%) and the FASTest^®^ CRYPTO-GIARDIA Strip (92%). All tests were easy to use and did not cross-react with *Eimeria* spp. oocysts. In addition to, 90 calves under one month old also tested by Hamedian et al. (2022) to check their immune responses to *Rotavirus* and *Bovine Coronavirus*. They used different diagnostic methods, and found that 8.89% of the calves were positive for *Rotavirus*, 14.44% for *Bovine Coronavirus*, and 2.22% had both infections at the same time. Ag ELISA was found to be the most accurate method for diagnosing RV and BCV, showing 100% sensitivity and 94.3% specificity compared to RT-PCR. The ICG test, while less accurate, still provided good results with 95% sensitivity and 94.3% specificity and showed good correlation and concordance with RT-PCR. The authors suggest confirming positive ICG results with Ag ELISA or RT-PCR to reduce mistakes in diagnoses on farms (Hamedian et al. 2022).

#### Molecular-based assays

##### Polymerase chain reaction (PCR)

The PCR technique is used to find and amplify a specific DNA sequence from a genome to billions of copies. It helps identify various intestinal pathogens. PCR tests are particularly effective for detecting hard-to-isolate viruses and slow-growing bacteria (Mohamed et al. 2017; Eldesoukey et al. 2022). These assays have many benefits compared to culture and other methods, including high specificity, sensitivity, speed, accuracy, and the ability to detect small amounts of target nucleic acid in samples (Saleh et al. 2022a). The presence of Enterotoxigenic *E. coli* (ETEC) K99 and *Salmonella* spp. in diarrheic newborn calves was studied by Younis et al. (2009) in Egypt. They collected fecal samples from 220 calves on nine farms across four areas. Using PCR, they found ETEC K99 in 10.36% of samples and *Salmonella* spp in 4.09%. The study identified links between ETEC K99 infection and factors like age, colostrum feeding, and co-infection with *Rotavirus* and *Coronavirus*. It concluded that PCR is useful for quickly screening these pathogens in calves with diarrhea. A study carried by Mohamed et al. (2017) on two Egyptian farms with calves suffering from diarrhea found that 76% of samples tested positive for enteric viruses, particularly *Rotavirus* (48%), Norovirus (24%), and Astrovirus (32%), using RT-PCR. This was the first report of bovine norovirus and astrovirus in Egypt. Additionally, Eldesoukey et al. (2022) examined the presence of Enteropathogenic *E. coli* (EPEC) in diarrheal calves, milk, and farm workers across three dairy farms using repetitive extragenic palindromic sequence-based PCR (REP-PCR). They found that 22.7% of diarrheic calves had EPEC, with certain strains showing high genetic similarity to those found in farm workers, suggesting possible zoonotic transmission. El-Seedy et al. (2016) studied the presence of *Salmonella* and *E. coli* in calves with diarrhea by examining stool samples from 127 calves under three months old. They found that 119 bacteria isolates (93.7%) were present, with *Salmonella* at 18.1% and *E. coli* at 75.6%. The serotyping of *Salmonella* showed that S. *Enteritidis* and *S. Typhimurium* were the most common, representing 60.9% and 30.4% respectively. *E. coli* was found in ten O-serogroups, with O26 and O103 being the most common (17.7% each). PCR tests indicated positive results for *Salmonella*, while 70% of *E. coli* serogroups had the ETEC virulent gene (K99). Additionally, Sultan et al. (2013) confirmed the presence of *Toxocara vitulorum* in cattle in El-Mahlla El-Kubra using molecular techniques, revealing that Egyptian strains are genetically (ITS-1 and 18 S ribosomal DNA) similar to those in other countries and closely related to zoonotic *Toxocara* species.

The infection rates of *Cryptosporidium* in buffaloes, cattle, and sheep were studied by Mahfouz et al. (2014). They tested rectal fecal samples of 466 buffaloes, 1697 cattle, and 120 sheep using modified Zeil-Neelsen acid-fast microscopy. The average infection was 1.29% in buffaloes, 7.07% in cattle, and 2.50% in sheep, with significant variations between calves and adults. PCR-RFLP analyses of small-subunit rRNA genes from positive specimens revealed the occurrence of *C. parvum* and *C. ryanae* in buffalo; *C. parvum*, *C. ryanae*, *C. bovis* and *C. andersoni* in cattle and only *C. xiaoi* in sheep. Genotypes distribution showed that *C. ryanae* was the dominant species (60.0%) followed by *C. parvum* (40.0%) in buffalo calves. Meanwhile, in cattle calves, *C. parvum* was the commonest species (74.23%) followed by *C. ryanae* (16.10%) and *C. bovis* (9.70%). Subtyping of *C. parvum* based on sequence analysis of the polymorphic 60 kDa glycoprotein gene locus showed the presence of subtypes IIdA20G1 and IIaA15G1R1 in both buffalo and cattle calves, addressing the potential role of calves in zoonotic cryptosporidiosis in Egypt.

Diarrheal fecal samples from calves aged 1-103 days from 23 farms in Ningxia, China were studied by Wang et al. (2023) using PCR. They looked for 15 common pathogens causing diarrhea in calves, including viruses, bacteria, and parasites. The most frequently found pathogens were *Cryptosporidium* (50.40%), *Bovine Rotavirus* (BRV) (23.18%), *E. coli* K99 (20.0%), and *Bovine Coronavirus* (BCoV) (11.82%). Other pathogens such as *Coccidia* (6.90%), *Bovine Astrovirus* (BoAstV) (5.46%), *Bovine Torovirus* (BToV) (4. 09%), and *Bovine Kobuvirus* (BKoV) (3.18%) were mostly identified in combinations with other infections. In addition to, Wei et al. (2021) carried out a study comparing two methods to test faecal samples from diarrheic calves for pathogens that cause neonatal calf diarrhea in China. They collected 69 faecal samples from diarrheic calves, and used PCR and a rapid test kit; Antigen Rapid BoviD-5 Ag Test Kit. The PCR method showed positive results for pathogens as follows: *C. parvum* (44.93%), Bovine *Rotavirus* A (36.23%), *Bovine Coronavirus* (17. 39%), and *Giardia lamblia* (13.04%). The rapid test kit showed slightly higher positivity rates: *C. parvum* (52.17%), *Bovine Rotavirus* A (31.88%), *Bovine Coronavirus* (28.98%), and *Giardia lamblia* (18.84%). Both methods did not detect *E. coli* K99. The overall positivity rates from PCR and the rapid test were 80.00% and 81.16%, respectively, showing no significant differences between the two methods. The results indicate that both methods can be used in clinics for diagnosing calf diarrhea. Inhibition of PCR reactions by components in fecal samples can affect DNA-based testing, requiring effective DNA extraction methods and controls to address this issue, which might lower sensitivity (Schrader et al. 2012).

##### Multiplex PCR (mPCR)

Traditionally, PCR and qPCR tests focused on detecting one pathogen have been common because they are easier to design and conduct (Saleh et al. 2022a). However, tests that can identify multiple infections at once, like duplex or multiplex assays, are increasingly popular (Chang et al. 2023). They use less sample volume and materials, lower costs, save time, and make the diagnostic process more efficient, especially in diagnosing diarrhea. *E. coli*-related diarrhoea in young calves using multiplex PCR was assessed by Abed and Menshawy (2019). They found enterotoxigenic *E. coli* (ETEC) and enteroaggregative *E. coli* (EAEC) in 70% of samples, and enterohemorrhagic *E. coli* (EHEC) in 30%, but no Shiga toxin-producing *E. coli* (STEC). A new multiplex quantitative PCR (qPCR) test called Enterit4Calves that helps find and measure pathogens causing diarrhea in calves was studied by Pansri et al. (2022). It looks for pathogens like *C. perfringens* type B and C, *Salmonella* Dublin, *E. coli* F5, *bovine Rotavirus*, *bovine Coronavirus*, *C. parvum*, and *Eimeria* in stool samples. The test showed an efficiency of 84% −103% and could detect 100–1000 copies of nucleic acids per sample. They correctly identified 42 strains of target bacteria with only one false positive reaction from 135 non-target bacteria. The novel qPCR method showed good performance under laboratory conditions and a fair to good agreement with current routine methods when used for testing of field samples (Pansri et al. 2022).

##### Real-time PCR (quantitative PCR or qPCR)

Real-time PCR is a method that uses fluorescent probes to identify DNA during the amplification process, making it faster and simpler than standard PCR. It employs two types of agents: specific fluorescent probes for certain DNA sequences and dyes like SYBR Green I and SYBR Gold, which attach to any double-stranded DNA. This method offers high sensitivity and specificity, reduces contamination risk, and is easy to use. It is also a quick alternative to traditional culture or immunoassays. TaqMan PCR, a type of real-time PCR, can amplify DNA from microorganisms found in complex biological samples (Arya et al. 2005; Espy et al. 2006). A new method was developed by Meng et al. (2024) using multiplex real-time fluorescence quantitative PCR to detect viruses that cause diarrhea in calves. This includes viruses like bovine torovirus, bovine enterovirus, bovine norovirus, bovine *Coronavirus*, Bovine Rotavirus, and *Bovine Viral Diarrhea* virus. The method is very sensitive and reliable, with detection limits between 1.91 copies/µL and 96.0 copies/µL for the different viruses, and shows strong effectiveness in amplification. This detection technology offers important insights into the occurrences of calf diarrhea viruses and could help inform future prevention and control strategies. Eighty two feces samples from calves aged 0–1 month were collected by Elsadek et al. (2022); they used qRT-PCR with specific primers to check for BRV and BCoV nucleic acids. They found that 14 samples (17. 1%) were positive for BRV and 22 samples (26. 8%) were positive for BCoV. Sixty nine fecal samples from newborn calves with diarrhea were examined by Golaviya et al. (2024) using different methods to detect *Bovine Rotavirus* A (BRVA). They used latex agglutination test (LAT), RNA electropherotyping (RNA-PAGE), and reverse transcription polymerase chain reaction (RT-PCR), finding BRVA detection rates of 39.13% for LAT, 20.30% for RNA-PAGE, and 37.70% for RT-PCR. The effectiveness of real-time reverse transcription recombinase polymerase amplification (RT-RPA) assays to detect BRVA in cattle was evaluated by Liu et al. (2022), achieving detection limits of 1. 4 × 10^2^ copies for real-time and 1. 4 × ^1^01 copies for lateral flow strip (LFS RT-RPA). In their study, the positive rates for BRVA in fecal samples were 45.52% using RT-RPA and 46.27% using lateral flow strip (LFS RT-RPA).The real-time RT-RPA and lateral flow strip (LFS RT-RPA) tests demonstrated a diagnostic specificity of 100% and sensitivities of 98. 39% and 100%, respectively. The kappa coefficients were 0.985 and 1.0. The authors found that these RT-RPA tests were highly specific, extremely sensitive, and easy to use. They showed great potential for quick detection of BRVA in settings with limited diagnostic resources, such as quarantine facilities and farms.

##### Digital polymerase chain reaction (dPCR)

**Fig. 1:**
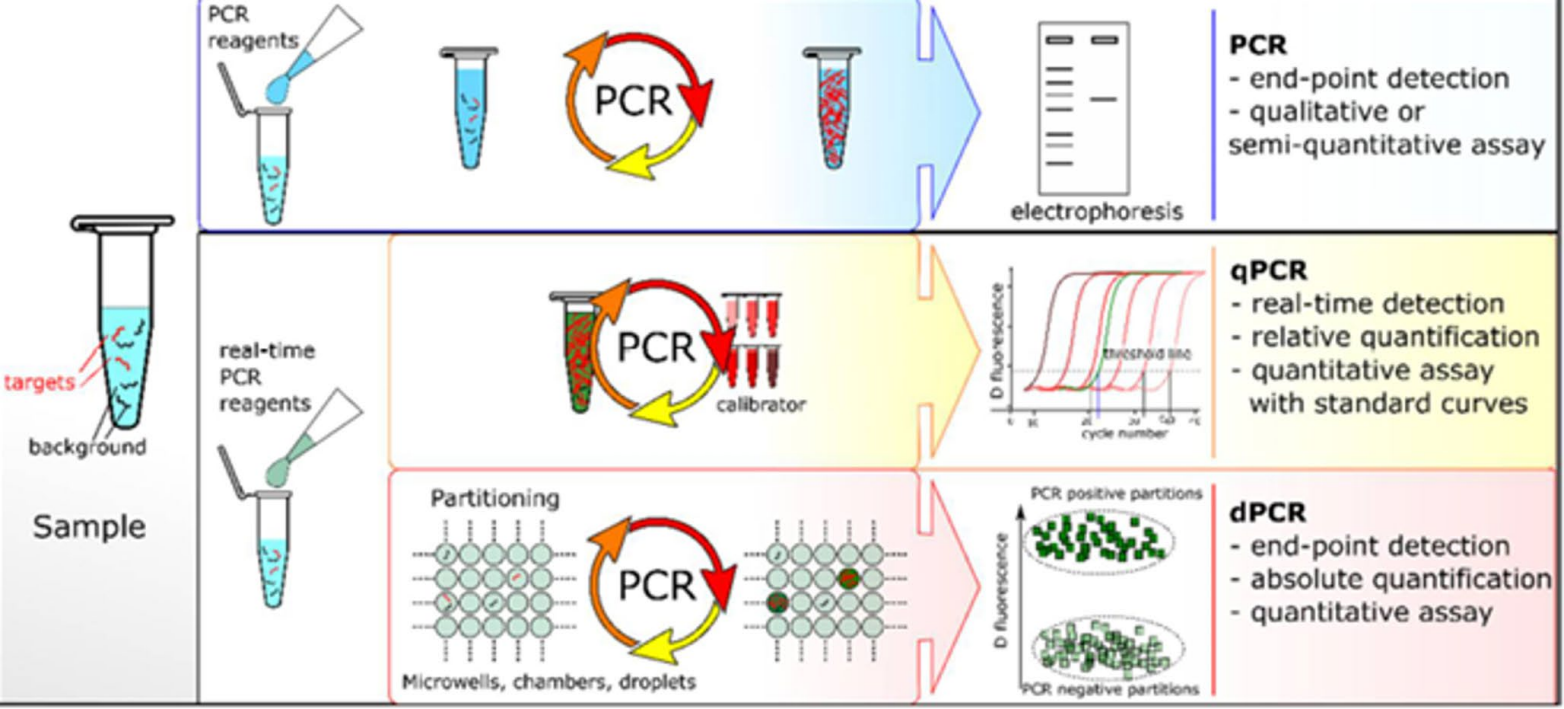
Comparison of PCR-based techniques (Quan et al. 2018)

Digital Polymerase Chain Reaction (dPCR) is a groundbreaking method for finding the exact amount of specific nucleic acids. It works by using the random distribution of molecules in separate divisions, which follow a Poisson distribution as seen in Fig. 1. Each division acts as an independent PCR microreactor, with partitions that contain target sequences detected through fluorescence. The number of PCR-positive partitions helps determine the concentration of the target sequence without needing calibration. Recent progress in microfluidics has greatly improved digital quantification methods (Cao et al. 2017).

The comparison of PCR-based approaches is presented in Fig. 1. Traditional PCR analyzes amplified products at the end using gel electrophoresis and fluorescent labeling. Both qPCR and dPCR use the same chemicals and labeling devices. In qPCR, the amount of DNA is measured during each cycle in real time, using a standard curve for accuracy. In dPCR, the sample is split into smaller parts, with some containing the target sequences. After PCR, the proportion of positive results in these parts helps calculate the concentration of the target sequence using Poisson’s statistics (Cao et al. 2017). The droplet digital PCR (ddPCR) method was improved by Chen et al. (2023) to detect bovine Enterovirus (BEV), *bovine Coronavirus* (BCoV), and *bovine Rotavirus* (BRV) in 82 calves with diarrhea. The detection limits for traditional qPCR were higher at 1,000 copies/µL, while the limits for ddPCR were lower at 2.7, 1, and 2.4 copies/µL for BEV, BCoV, and BRV, respectively. The dual ddPCR method identified BCoV, BRV, and co-infection rates of 18.29%, 14.63%, and 6.1%, while standard qPCR found lower rates of 10.98%, 12.2%, and 3.66%. Overall, ddPCR proved to be more effective than traditional qPCR in detecting these viral infections.

##### Loop-mediated isothermal amplification (LAMP) assay

The LAMP method quickly produces a large amount of DNA in less than an hour using only one kind of enzyme and 4–6 specific primers, without requiring extra reagents (Bodulev and Sakharov 2020). One key benefit of LAMP over PCR is that it reduces the risk of contamination by performing the entire process in one reaction tube at a constant temperature. LAMP is known for being highly accurate and sensitive in amplifying nucleic acids (Pang et al. 2019). LAMP can detect not only bacterial and viral infections but also harmful protozoans like *Cryptosporidium* (Karakavuk et al. 2024). It is quick, easy to to apply, and works at one temperature, making it suitable for field use. LAMP does not need complicated lab equipment or highly trained personnel, requiring only a water bath or thermal block to keep a stable temperature during the process (Nagamine et al. 2002). The LAMP test used to find *Cryptosporidium spp.* in 127 calf feces by Karakavuk et al. (2024), achieving 100% sensitivity and 97. 4% specificity when compared to Real-Time PCR. The test did not react with other parasites or bacteria, and diagnosis can be done just visually, without equipment.

##### DNA microarray and whole-genome sequencing

Immunological and molecular assays can detect gut pathogens but struggle with issues like antibody stability, high PCR costs, and cross-reactivity. DNA microarray and whole-genome sequencing offer detailed genetic information. DNA microarray technology is an important method that enables the testing of many DNA samples at once. It allows multiple DNA probes to attach to their matching DNA sequences during one experiment. This technique helps researchers study genes and their functions more efficiently. Initially, it was used for gene expression analysis, allowing researchers to study the expression patterns of thousands of genes simultaneously (Donatin and Drancourt 2012). Microarrays have many uses now, especially in comparing genomes and analyzing sequences. They are effective for finding genetic differences in closely related samples and for diagnostics. A key benefit of microarrays is their ability to multiplex, which allows for the detection of multiple enteropathogens in stool samples. Chips can identify certain harmful bacteria, like enterotoxinogenic *E. coli*, enterohemorrhagic *E. coli*,* S. enterica* serovar Enteritidis, *S. enterica* serovar *Typhimurium*, and *Salmonella enterica* (Wang et al. 2010). However, their high cost and technical challenges limit their use. Whole-genome sequencing (WGS) is a complex method that uses automated technology to analyze the complete genomes of enteric pathogens. Unlike DNA microarrays, which focus on specific genes, WGS looks at all bacterial DNA and creates full genome sequences. These sequences can be compared to existing databases like NCBI and CFSAN-FDA for analysis and identification. Such databases support comparative genomics, helping researchers track pathogen evolution, recognize antimicrobial resistance, and monitor outbreaks. The occurrence and molecular characteristics of Bovine Viral Diarrhoea, Rota, and Corona viral diseases in calves across various Egyptian regions, including Kafr El Sheikh, El Menofyia, Sharkia, Bahaira, and El-Gharbia were studied by Lotfy et al. (2020). Their findings showed that the prevalence of these diseases did not significantly differ based on the age or sex of the calves, although infections were more common in female young calves. Some of the positive samples were sequenced and compared to other strains in GenBank. Investigations showed that the Egyptian BVD strain is closely related to BVD strains from Argentina and Colombia. Bovine *Rotavirus* (BRV) is similar to strains from South Korea and Turkey. Bovine *Coronavirus* (BCoV) is related to a strain isolated from France. Fifty fecal samples from calves with diarrhea up to 3 months old over a year were collected by Abouelyazeed et al. (2019). Their analysis showed a perfect match in sequences with BCoV strains like BCV Menofyia, FRA8, and FRA10, as well as a 99. 6% match with strains FRA03 and FRA05 from the gene Bank database.

#### Nanotechnology-based assays

Nanotechnology-based platforms are changing how we diagnose diseases. By using tiny particles called nanoparticles, these platforms improve the ability to detect and identify different health conditions more accurately and sensitively. This new approach enhances traditional detection methods significantly that include ELISA, lateral flow assays, IFA, and PCR (Wang et al. 2019; Ali et al. 2021; Ekinci et al. 2022). Nanomaterials make assays more accurate and effective, allowing for earlier disease detection and genetic profiling. They offer simpler, faster, and cheaper testing methods. Oligonucleotide nanoparticles (NPs) have been created for molecular detection, eliminating the need for traditional nucleic acid amplification. The study by Weigum et al. (2013) used gold nanoparticles to detect *Cryptosporidium parvum* oocysts. This method shows color changes when RNA is present, allowing detection of 670 oocysts per µL in spiked stool samples. It is anticipated that such pre-processing techniques will enhance the capability of the DNA/RNA-linked AuNP assay for rapid, amplification-free detection of diarrhea-causing intestinal protozoans with high sensitivity and specificity. These developments offer a quick and cost-effective way to diagnose diseases in low-resource areas. Biosensor technology is important for modern diagnostics, especially for quick testing. Electrochemical biosensors are becoming popular because they are fast, sensitive, and specific. These sensors turn biological responses into electrical signals that help detect specific pathogens or biomarkers (Perumal and Hashim 2014; Da Silva et al. 2017). Using nanomaterials such as graphene derivatives, carbon nanotubes, and various nanoparticles increases their effectiveness. Nanomaterials enhance the surface area, conductivity, and sensitivity of sensors, allowing for much lower detection limits than traditional biosensors that use only antibodies or molecular probes. Nano-Electrochemical biosensors applied to *Salmonella* and *E. coli* O157:H7 diagnostics show that combining electrochemical biosensors with nanotechnology offers a fast, reliable, and highly sensitive method for detecting pathogens (Razmi et al. 2020). This approach is ideal for clinical and environmental use and could significantly improve microbial diagnostics by enabling quicker and more accurate detection. A sensitive immunosensor to detect *Cryptosporidium parvum* was developed by Thiruppathiraja et al. (2011). It uses an enzyme-amplified sandwich structure with dual-labeled gold nanoparticles on an ITO electrode. The sensor can detect as low as 3 oocysts per milliliter.

### Hematological and serum biochemical analyses

Hematological and serum biochemistry analyses are essential for identifying the causes of diarrhea in calves and determining appropriate treatments. Haematological and metabolic changes including decrease in glucose concentration increase in urea and creatinine concentration in blood, loss of carbohydrates and accumulation of organic acid, which conduce to the appearance of metabolic acidosis in calves with dihrea (Dratwa et al. 2012). Hyponatremia, hyperkalemia and hypochloremia are usually observed together with an increasing degree of dehydration in diarrheic calves; Hyperkalemia was also seen in diarrhetic calves in some studies, and it had a direct relationship with disturbances in the acid-base balance (Dratwa et al. 2012). Changes in neutrophil levels like neutropenia can indicate severe diarrhea. Also, a high packed cell volume (PCV) shows dehydration and signals the need for quick action (Tothova et al. 2016). Sixty two calves with diarrhea, aged 1 to 6 months were eximined by Abed et al. (2020), finding increases in hemoglobin, packed cell volume, and white blood cells. There were significant rises in globulin, potassium, and phosphorus, while albumin and sodium levels dropped. In addition to, diarrheic calves (7 days till 6 months) had higher levels of pro-inflammatory markers like IL-6 and haptoglobin, but lower levels of total antioxidant capacity and IgA (Saleh et al. 2022a; Sayar and Keles 2024); these findings underscore *E. coli*,* Salmonella*, and *Cryptosporidium* as key causes of calf diarrhea. In contrast to, Barua et al. (2018) found that there were no significant differences in blood tests of diarrheic calves, whether they had *Rotavirus* or not, except for a higher lymphocyte count of 75.58%, indicating a virus cause. On the other hand, the neutrophil count was lower in diarrheic calves as compared with the normal values and the average mean value appeared as 15.41% and 19.48% in *Rotavirus* positive and negative cases, respectively.In *Rotavirus*-positive cases, chloride levels were higher at 115.05%, indicating hyperchloremia in those calves (under the age of 45 days) (Ekinci et al. 2024). Hyperchloremia is a typical consequence of the occurred metabolic acidosis resulting from diarrhea caused by *Rotavirus* as well as other etiological agent. hematological and biochemical changes from *Eimeria* and *Toxocara* infections in calf diarrhea (7 days till 6 months) showed significant increases in red cell count, hemoglobin, packed cell volume, and total leukocyte count. Enzymatic activity levels of ALT, AST, ALP, GGT, amylase, and lipase were notably elevated in the diseased group. Serum glucose, urea, BUN, and creatinine levels increased significantly, while total protein, albumin, triglyceride, and electrolytes (Na, K, and Cl) decreased (Saleh et al. 2022b).

### Postmortem examination

**Fig. 2:**
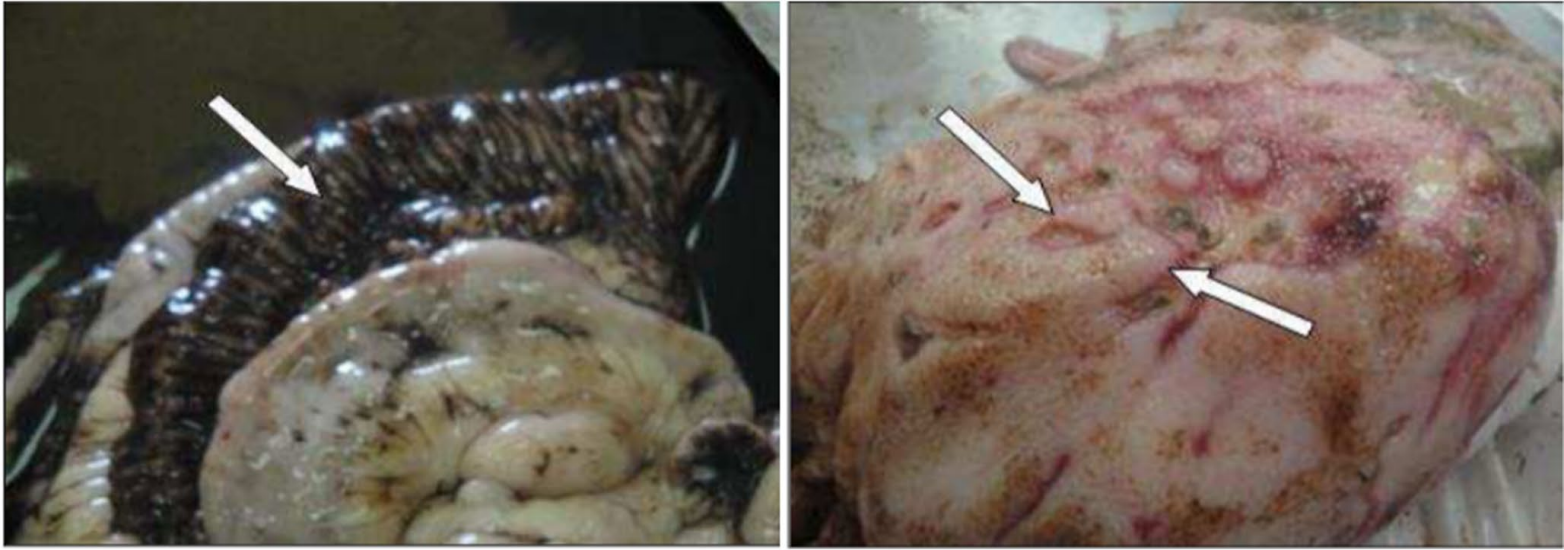
Gross alterations in the digestive tract of calves that died as a result of *Clostridium perfringens* infection, demonstrating blackish mucosa in the ileum and multiple ulcers in Abomasum (Hamouda et al. 2014)

**Fig. 3:**
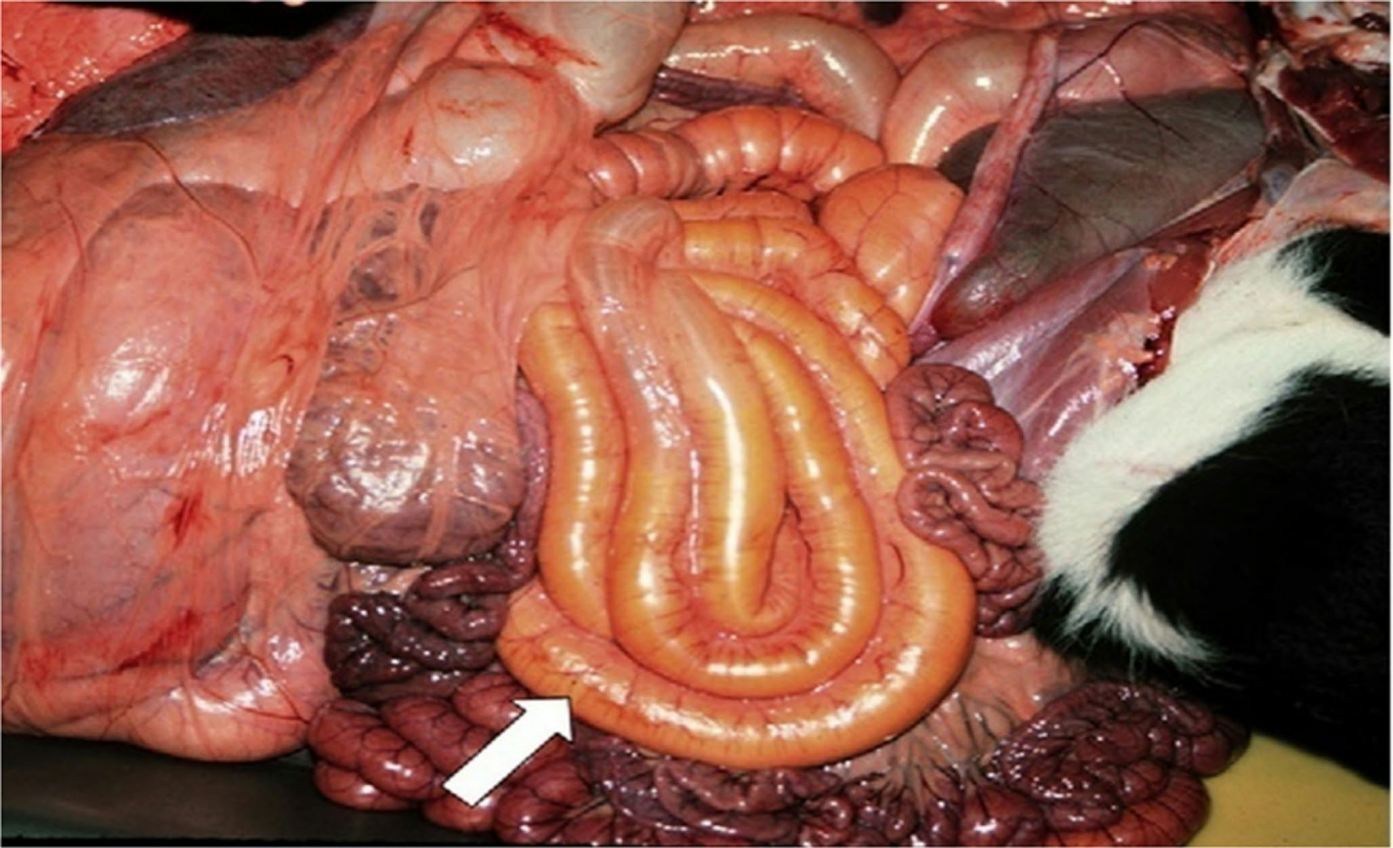
*E. coli* (K99) causes spiral colon bulging and watery yellow contents as shown by the arrow (Blanchard 2012)

Postmortem examination is important for veterinarians to check intestinal issues and identify pathogens like *Clostridium perfringens*. This method is sometimes the only way to confirm a diagnosis. Clostridial diseases progress rapidly and sudden death is often the first and the only sign of disease (Simpson et al. 2018). *C.perfringens* type C causes necrotic enteritis in newborncalves. Calves are suddenly depressed, weak, and maybe distended or show abdominal pain. If diarrhea develops, it may have blood and tissue streaks (Muktar et al. 2015). Affected calves may die before they develop diarrhea. *Clostridium perfringens* can cause serious damage in calves as shown in Fig. 2, leading to multiple ulcers in Abomasum and blackish mucosa in ileum (Hamouda et al. 2014). *E. coli* (K99) infection can severely affect calves, causing dehydration and electrolyte imbalances before diarrhea occurs (Blanchard 2012). This infection also results in noticeable changes in the colon, as shown in Fig. 3. Necropsy is most helpful when done shortly after death because important findings can disappear quickly due to the body’s natural processes. Therefore, it is essential to carry out a postmortem examination as soon as possible to get accurate diagnostic information. Due to the initiation of postmortem autolysis within approximately five minutes of death, prompt necropsy is imperative (McConnel et al. 2019).

## Conclusion

Diarrhoea in newborn calves can become quickly serious, so it’s important to diagnose it fast. This helps veterinarians provide the right treatments on time. The clinical history is a key for understanding, preventing, and treating diarrhea in newborns. It should include vaccination records, age, and colostrum consumption, how long diarrhoea has lasted, and how many animals have been affected or died in the herd. F5 (K99) *E. coli* is a common cause of diarrhoea in animals under 7 days old, but *Cryptosporidium* and *Coccidia* are found later due to their prepatent phases. Blood in calves’ feces under 30 days old is usually linked to *Salmonella* or *Coronavirus*, with less common links to *Cryptosporidium* and *Clostridium*. Many pathogens can cause diarrhea, so lab testing is essential for a correct diagnosis. Bacterial culturing is a key method for isolating and identifying bacterial pathogens from fecal and intestinal samples. Viral isolation has important benefits. It can confirm the presence of a harmful virus in clinical samples. The isolated virus can then be used for research on genetics, immune response, and how the virus causes disease. It also helps in creating diagnostic kits and vaccines. Advanced immunological and molecular methods like PCR, real-time PCR, multiplex PCR, LAMP, digital PCR, immunochromatographic tests, and nano-electrochemical biosensors provide highly sensitive and specific diagnoses. These techniques are particularly useful for detecting viruses that are difficult to isolate in labs and bacteria that require lengthy development time. Real-time PCR, multiplex PCR, isothermal amplification tests (like LAMP) are better than standard methods for diagnosing diarrhea in newborn animals. They are very accurate, sensitive, fast, and can test many samples at once and. The ability to detect several parasites at once with a single test, like duplex or multiplex assays, is valuable. It lowers the amount of sample needed, cuts costs for materials and reagents, saves time, simplifies the testing process, and makes screening easier. Immunochromatographic assays which are important, rapid, less expensive and easy to perform field tests. However, PCR Real-time PCR, multiplex PCR, isothermal amplification tests (like LAMP) are still more sensitive and specific.

## Data Availability

No datasets were generated or analysed during the current study.

## References

[CR1] Abdou NEMI, Majeed QAH, El-Azazy OME et al (2021) Risk factors of diarrhea in small ruminants in Kuwait. Iran J Vet Res 22:146–149. 10.22099/ijvr.2021.38092.5546 34306113 10.22099/ijvr.2021.38092.5546 PMC8294814

[CR2] Abed AH, Menshawy AMS (2019) Escherichia coli neonatal calf diarrhea in middle Egypt: prevalence, phenotypes, genotypes and pathotypes. World J Vet Sci 7:14–23. 10.12970/2310-0796.2019.07.04

[CR3] Abed ZEM, Sulaiman YAM, Khalaf HY (2020) Evaluation of some biochemical and hematological parameters for changes associated with diarrhea in calves. Tikrit J Pure Sci 25:7–9. 10.25130/tjps.v25i1.205

[CR4] Abouelyazeed EA, Yanni MI, Moussa et al (2019) Advanced virologic detection of coronavirus from infected calves. Anim Health Res J 7:445–450

[CR5] Abu El Ezz NMT, Khalil FAM, Abd El-Razik KA (2020) Molecular epidemiology of cryptosporidiosis in pre-weaned cattle calves in Egypt. Bulg J Vet Med 23:112–120. 10.15547/bjvm.2167

[CR6] Akilesh S, Nicosia RF, Alpers CE et al (2021) Characterizing viral infection by electron microscopy. Am J Path 191:222–227. 10.1016/j.ajpath.2020.11.003 33227297 10.1016/j.ajpath.2020.11.003 PMC7678435

[CR7] Algammal AM, El-Kholy AW, Riad EM et al (2020) Genes encoding the virulence and the antimicrobial resistance in enterotoxigenic and Shiga-Toxigenic E. coli isolated from diarrheic calves. Toxins 12:383. 10.3390/toxins12060383 32532070 10.3390/toxins12060383 PMC7354582

[CR8] Ali A, Ijaz M, Khan YR et al (2021) Role of nanotechnology in animal production and veterinary medicine. Trop Anim Health Prod 53:508. 10.1007/s11250-021-02951-5 34626253 10.1007/s11250-021-02951-5

[CR9] Altuğ N, Yüksek N, Özkan C et al (2013) Rapid etiological diagnosis of neonatal calf diarrhoea with immunochromatographic test kits. J Faculty Vet Med Yüzüncü Yil University 24:123–128. ISSN: 1017–8422; e-ISSN: 1308–3651

[CR10] Arya M, Shergill IS, Williamson M et al (2005) Basic principles of real-time quantitative PCR. Expert Rev Mol Diagn 5:209–219. 10.1586/14737159.5.2.209 15833050 10.1586/14737159.5.2.209

[CR11] Ata EB, Nasr SM, Mohamed AM et al (2020) Bacteriological, hematological and biochemical diagnostic studies on diarrheic Arabian horse foals caused by enterobacterial infections. Adv Anim Vet Sci 8:412–421. 10.17582/journal.aavs/2020/8.4.412.421

[CR12] Ates O, Yesilbag K (2023) Characterization of bovine rotavirus isolates from diarrheic calves in Türkiye. Mol Biol Rep 50:3063–3071. 10.1007/s11033-022-08169-4 36689052 10.1007/s11033-022-08169-4 PMC9870195

[CR13] Athanassious R, Marsolais G, Assaf R (1994) Detection of bovine coronavirus and type A rotavirus in neonatal calf diarrhea and winter dysentery of cattle in Quebec: evaluation of three diagnostic methods. Can Vet J 35:163–169 PMID: 8055431; PMCID: PMC1686320 8055431 PMC1686320

[CR14] Barua SR, Rakib TM, Shubhagata D et al (2018) Hematological and serological changes in neonatal diarrheic calves infected with bovine rotavirus. Multidisciplinary Adv Veterinary Sci 2(3):356–366

[CR15] Berber E, Çanakoğlu N, Sözdutmaz I et al (2021a) Seasonal and age-associated pathogen distribution in newborn calves with diarrhea admitted to ICU. Vet Sci 8:128. 10.3390/vetsci8070128 34357920 10.3390/vetsci8070128 PMC8310227

[CR16] Berber E, Şimşek E, Çanakoğlu N et al (2021b) Newly identified Cryptosporidium parvum virus-1 from newborn calf diarrhoea in Turkey. Transbound Emerg Dis 68:2571–2580. 10.1111/tbed.13929 33207084 10.1111/tbed.13929

[CR17] Blanchard PC (2012) Diagnostics of dairy and beef cattle diarrhea. Vet clin N AM-Food A. 28:443–464. 10.1016/j.cvfa.2012.07.002 10.1016/j.cvfa.2012.07.002 PMC7127268 23101670

[CR18] Bodulev OL, Sakharov IY (2020) Isothermal nucleic acid amplification techniques and their use in bioanalysis. Biochem (Mosc) 85:147–166. 10.1134/S0006297920020030 10.1134/S0006297920020030 PMC7223333 32093592

[CR19] Caffarena RD, Casaux ML, Schild CO et al (2021) Causes of neonatal calf diarrhea and mortality in pasture-based dairy herds in Uruguay: a farm-matched case-control study. Braz J Microbiol 52:977–988. 10.1007/s42770-021-00440-3 33575990 10.1007/s42770-021-00440-3 PMC7877513

[CR20] Cao L, Cui X, Hu J et al (2017) Advances in digital polymerase chain reaction (dPCR) and its emerging biomedical applications. Biosens Bioelectron 90:459–474. 10.1016/j.bios.2016.09.082 27818047 10.1016/j.bios.2016.09.082

[CR21] Catroxo MHB, Miranda LB, Lavorenti A et al (2010) Detection of coronavirus in capybaras (Hydrochoeris hydrochaeris) by transmission electron microscopy in Sao paulo, Brazil. Int J Morphol 28:549–555. 10.4067/S0717-95022010000200035

[CR22] Celik BA, Celik OY, Ayan A et al (2022) A survey of Toxocara vitulorum in Anatolian water buffaloes (Bubalis Bubalis) in Diyarbakir, Turkey. Assiut Vet Med J 68:90–96. 10.21608/avmj.2022.149998.1072

[CR23] Chang L, Li Y, Cai Y, CLi (2023) Establishment of multiplex PCR for detection of calf diarrhea associated virus and analysis of its clinical infection status Pakistan. J Zool 55:2501–3000. 10.17582/journal.pjz/20220701010749

[CR24] Chen J, Li D, Xu Y et al (2023) Establishment and application of multiplex droplet digital polymerase chain reaction assay for bovine enterovirus, bovine coronavirus, and bovine rotavirus. Front Vet Sci 10:1157900. 10.3389/fvets.2023.1157900 37771940 10.3389/fvets.2023.1157900 PMC10523346

[CR25] Cho YI, Han JI, Wang C et al (2013) Case-control study of microbiological etiology associated with calf diarrhea. Vet Microbiol 166:375–385. 10.1016/j.vetmic.2013.07.001 10.1016/j.vetmic.2013.07.001 PMC7117237 23886509

[CR26] Constable PD (2004) Antimicrobial use in the treatment of calf diarrhea. J Vet Intern Med 18:8–17. 10.1892/0891-6640(2004)18%3C;8:auitto%3E;2.0.co;2 10.1111/j.1939-1676.2004.tb00129.x PMC7197454 14765726

[CR27] Constable PD, Walker PG, Morin DE, Foreman JH (1998) Clinical and laboratory assessment of hydration status of neonatal calves with diarrhea. J Am Vet Med Assoc 212:991–996 9540870

[CR28] Coura FM, Freitas MD, Ribeiro J et al (2015) Longitudinal study of Salmonella spp., diarrheagenic Escherichia coli, rotavirus, and coronavirus isolated from healthy and diarrheic calves in a Brazilian dairy herd. Trop Anim Health Prod 47:3–11. 10.1007/s11250-014-0675-5 25311440 10.1007/s11250-014-0675-5 PMC7089331

[CR29] Cruvinel LB, Ayres H, Zapa DMB et al (2020) Prevalence and risk factors for agents causing diarrhea (Coronavirus, rotavirus, Cryptosporidium spp., Eimeria spp., and nematodes helminthes) according to age in dairy calves from Brazil. Trop Anim Health Prod 52:777–791. 10.1007/s11250-019-02069-9 31591674 10.1007/s11250-019-02069-9 PMC7089087

[CR30] Da Silva ET, Souto DE, Barragan JT et al (2017) Electrochemical biosensors in point-of-care devices: recent advances and future trends. Chem ElectroChem 4:778–794. 10.1002/celc.201600758

[CR31] Dahmani H, Ouchene N, Dahmani A et al (2020) First report on *Cryptosporidium parvum*, *Escherichia coli* K99, *rotavirus* and *coronavirus* in neonatal lambs from north-center region, Algeria. Comp Immunol Microbiol Infect Dis 73:101567. 10.1016/j.cimid.2020.101567 33157428 10.1016/j.cimid.2020.101567 PMC7580686

[CR32] Daly R, Rotert L (2007) Clostridium perfringens Infections in baby calves. Extension Extra. Paper 397. http://openprairie.sdstate.edu/extension_extra/397

[CR33] Davoudi Y, Nourmohammadzadeh F, Abdollahpour G, Nouri A, Nowrouzian I (2014) The prevalence of coronavirus in fecal samples of neonatal calf diarrhea using electron microscopic examination. Iran J Vet Med 8:85–89. 10.22059/IJVM.2014.51405

[CR34] Diaz P, Navarro E, Remesar S et al (2021) The Age-Related Cryptosporidium species distribution in asymptomatic cattle from North-Western SPAIN. Animal (Basel) 11:256. 10.3390/ani11020256 10.3390/ani11020256 PMC7909547 33498538

[CR35] Donatin E, Drancourt M (2012) DNA microarrays for the diagnosis of infectious diseases. Med Mal Infect 42:453–459. 10.1016/j.medmal.2012.07.017 23058632 10.1016/j.medmal.2012.07.017 PMC7127767

[CR36] Dratwa CA, Herosimczyk A, Lepczynski A, Skrzypczak IF (2012) Calves with diarrhea and a water-electrolyte balance. Medycyna Wet 68(1):5–8

[CR37] Dryden MW, Payne PA, Ridley R, Smith V (2005) Comparison of common fecal flotation techniques for the recovery of parasite eggs and oocysts. Vet Ther 6:15–28 15906267

[CR38] Ekinci GG, Tüfekçi E, Onmaz AC et al (2022) Investigation of the prevalence of major enteropathogens in neonatal diarrheic calves brought to Erciyes university animal hospital between 2019–2021 years. J Fac Veterinary Med Erciyes Univ 19:113–122

[CR39] Ekinci G, Tüfekçi E, Cissé Y et al (2024) Chloride and lactate as prognostic indicators of calf diarrhea from eighty-nine cases. J Vet Sci 25:e38. 10.4142/jvs.23155 38834508 10.4142/jvs.23155 PMC11156601

[CR40] El Seedy FR, Abed AH, Yanni HA, Abd El-Rahman SAA (2016) Prevalence of Salmonella and E. coli in neonatal diarrheic calves. Beni-Suef Univ J Basic Appl Sci 5:45–51. 10.1016/j.bjbas.2015.11.010 32363209 10.1016/j.bjbas.2015.11.010 PMC7185456

[CR41] Eldesoukey IE, Elmonir W, Alouffi A et al (2022) Multidrug-Resistant enteropathogenic Escherichia coli isolated from diarrhoeic calves, milk, and workers in dairy farms: a potential public health risk. Antibiot (Basel) 11:999. 10.3390/antibiotics11080999 10.3390/antibiotics11080999 PMC9332572 35892389

[CR42] Elhady AM, El-Azzouny MM, Khadra SH (2020) Factors affecting calf enteritis infection caused by Salmonellae and Escherichia coli. Assiut Vet Med J 66:21–43. 10.21608/avmj.2020.166376

[CR43] Elsadek E, Elshehidy M, Shahein M et al (2022) Molecular characterization of bovine rotaviruses and coronaviruses in diarrheic calves in Egypt (2014–2019). Zagazig Vet J 50:320–334. 10.21608/zvjz.2022.179675.1197

[CR44] Espy MJ, Uhl JR, Sloan LM et al (2006) Real-time PCR in clinical microbiology: applications for routine laboratory testing. Clin Microbiol Rev 19:165–256. 10.1128/CMR.19.1.165-256.2006 16418529 10.1128/CMR.19.1.165-256.2006 PMC1360278

[CR45] Fouad EA, Ramadan RM, Mohamed AM, Khalifa MM (2024) Prevalence of bacteriological and parasitological causes of diarrheic calves in middle Egypt. J Adv Vet Res 14:276–281. https://advetresearch.com/index.php/AVR/article/view/1534

[CR46] Gallagher JR, Kim AJ, Gulati NM, Harris AK (2019) Negative-stain transmission electron microscopy of molecular complexes for image analysis by 2D class averaging. Curr Protoc Microbiol 54:e90. 10.1002/cpmc.90 31518065 10.1002/cpmc.90 PMC6746251

[CR47] Garaicoechea L, Bok KL, Jones R, Combessies G (2006) Molecular characterization of bovine rotavirus circulating in beef and dairy herds in Argentina during a 10-year period (1994–2003). Vet Microbiol 118:1–11. 10.1016/j.vetmic.2006.06.004 16982159 10.1016/j.vetmic.2006.06.004

[CR48] Garro CJ, Morici GE, Tomazic ML et al (2021) Occurrence of Cryptosporidium and other enteropathogens and their association with diarrhea in dairy calves of Buenos Aires province, Argentina. Vet Parasitol Reg Stud Rep 24:100567. 10.1016/j.vprsr.2021.100567 10.1016/j.vprsr.2021.100567 34024383

[CR49] Ghazy AA, Abdel-Shafy S, Shaapan RM (2015) Cryptosporidiosis in animals and man: 2. Diagnosis. Asian J Epidemiol 8:84–103. 10.3923/aje.2015.84.103

[CR50] Golaviya A, Mathakiya R, Jakhesara S, Koringa P (2024) Determining genetic diversity of prevalent G and P genotype of bovine rotavirus A from neonatal calves of Gujarat, India. J Vet Sci 25:e55. 10.4142/jvs.24124 10.4142/jvs.24124 PMC11291431 39083207

[CR51] Guy JS (2015) Isolation and propagation of coronaviruses in embryonated eggs. Methods Mol Biol 1282:63–71. 10.1007/978-1-4939-2438-7_7 25720472 10.1007/978-1-4939-2438-7_7 PMC7121728

[CR52] Hamedian AM, Zakian A, Azimpour S et al (2022) Evaluation of diagnostic methods for the detection of bovine coronavirus and rotavirus in feces of diarrheic calves. J Hell Vet Med Soc 73:3951–3960. 10.12681/jhvms.23704

[CR53] Hamouda M, Al–hizab F, Fouda T, Fayez M (2014) Implication of clostridium perfringens type A in young calves. Res J Vet Pract 2:9–12

[CR54] Icen H, Arserim NB, Işik N et al (2013) Prevalence of four enteropathogens with immunochromatographic rapid test in the feces of diarrheic calves in East and Southeast of Turkey. Pak Vet J 33:496–499

[CR55] Ismael E, Kadry M, Hamza DA (2019) The occurrence of Clostridium difficile in different animal species in Egypt. Inter J Vet Sci 8:138–142

[CR56] Izzo MM, Kirkland PD, Gu X et al (2012) Comparison of three diagnostic techniques for detection of rotavirus and coronavirus in calf faeces in Australia. Aust Vet J 90:122–129. 10.1111/j.1751-0813.2011.00891.x 22443326 10.1111/j.1751-0813.2011.00891.x PMC7159673

[CR57] Jessop E, Li L, Renaud DL et al (2024) Neonatal calf diarrhea and gastrointestinal microbiota: etiologic agents and microbiota manipulation for treatment and prevention of diarrhea. Vet Sci 11:108. 10.3390/vetsci11030108 38535842 10.3390/vetsci11030108 PMC10975090

[CR58] Ji CY, Feng YQ, Sun RN et al (2023) Development of a multienzyme isothermal rapid amplification and lateral flow dipstick combination assay for bovine coronavirus detection. Front Vet Sci 9:1059934. 10.3389/fvets.2022.1059934 36686176 10.3389/fvets.2022.1059934 PMC9845563

[CR59] Karakavuk M, Can H, Can S et al (2024) Development of a Rapid-Crypto colorimetric LAMP test to detect cryptosporidiosis in feces of newborns calves. Acta Parasitolo 69:691–699. 10.1007/s11686-023-00791-x 10.1007/s11686-023-00791-x PMC11001726 38358452

[CR60] Kassem IK, Magouz A, Desouky AY, Hagag MF (2017) Isolation and identification of rotavirus infection in diarrheic calves at El Gharbia Governorate. Global Vet 18:178–182. 10.5829/idosi.gv.2017.178.182

[CR61] Keles İ, Ekinci G, Tüfekçi E et al (2022) Etiological and predisposing factors in calves with neonatal diarrhea: a clinical study in 270 case series. Kafkas Univ Vet Fak Derg 28:315–326. 10.9775/kvfd.2021.26981

[CR62] Khurana S, Chaudhary P (2018) Laboratory diagnosis of cryptosporidiosis. Trop Parasitolo 8:2–7. 10.4103/tp.TP_34_17 10.4103/tp.TP_34_17 PMC5991046 29930899

[CR63] Lee N, Choi SW, Chang HJ, Chun HS (2018) Rapid detection of Escherichia coli O157:H7 in fresh lettuce based on localized surface plasmon resonance combined with immunomagnetic separation. J Food Prot 81:713–718. 10.4315/0362-028X.JFP-17-338 29611731 10.4315/0362-028X.JFP-17-338

[CR64] Lichtmannsperger K, Freudenthaler K, Hinney B et al (2022) Evaluation ofimmunochromatographic point-of-care tests for the detection of calf diarrhoea pathogens in faecal samples. Wien Tierärztl Monat– Vet Med Austria 109:Doc11

[CR65] Liu Y, Liu L, Wang J et al (2022) Rapid detection of bovine rotavirus a by isothermal reverse transcription recombinase polymerase amplification assays. BMC Vet Res 18:339. 10.1186/s12917-022-03437-8 36076203 10.1186/s12917-022-03437-8 PMC9453720

[CR66] Lorenz I (2004) Investigations on the influence of serum D-lactate levels on clinical signs in calves with metabolic acidosis. Vet J 168:323–327. 10.1016/j.tvjl.2003.10.021 15501151 10.1016/j.tvjl.2003.10.021

[CR67] Lotfy A, Selim A, Ibrahim AM, Salem S (2020) Seroprevalence and molecular characterization of bovine viral diarrhea, Rota and corona viruses in neonatal cattle and buffalo calves in some governorates in Egypt. Benha Vet Med J 38:5–9. 10.21608/bvmj.2020.24852.1171

[CR68] Mahfouz ME, Mira N, Amer S (2014) Prevalence and genotyping of Cryptosporidium spp. in farm animals In Egypt. J Vet Med Sci 76:1569–1575. 10.1292/jvms.14-0272 25649937 10.1292/jvms.14-0272 PMC4300370

[CR69] Mainar-Jaime RC, Andres S, Vico JP et al (2013) Sensitivity of the ISO 6579:2002/amd 1:2007 standard method for detection of Salmonella spp. on mesenteric lymph nodes from slaughter pigs. J Clin Microbiol 51:89–94. 10.1128/JCM.02099-12 23100334 10.1128/JCM.02099-12 PMC3536256

[CR70] Makwana P, Irshadullakhan K, Dhruv D (2020) Isolation of bovine rotavirus in MDBK cell line from diarrhoeic calves of Navsari district. Pharma Innov 9:222–225. 10.22271/tpi

[CR71] Manuja BK, Prasad M, Gulati BR et al (2010) Comparative efficacy of immunological, molecular and culture assays for detection of group A rotavirus from faecal samples of buffalo (Bubalus bubalis) calves. Trop Anim Health Prod 42:1817–1820. 10.1007/s11250-010-9642-y 20607400 10.1007/s11250-010-9642-y

[CR72] Martella V, Catella C, Capozza P et al (2020) Identification of astroviruses in bovine and buffalo calves with enteritis. Res Vet Sci 131:59–68. 10.1016/j.rvsc.2020.04.010 32304933 10.1016/j.rvsc.2020.04.010 PMC7195147

[CR73] McConnel CS, Nelson DD, Burbick CR et al (2019) Clarifying dairy calf mortality phenotypes through postmortem analysis. J Dairy Sci 102:4415–4426. 10.3168/jds.2018-15527 30879809 10.3168/jds.2018-15527 PMC7094407

[CR74] Meganck V, Hoflack G, Opsomer G (2014) Advances in prevention and therapy of neonatal dairy calf diarrhoea: a systematical review with emphasis on colostrum management and fluid therapy. Acta Vet Scand 56:75. http://www.actavetscand.com/content/56/1/75 25431305 10.1186/s13028-014-0075-x PMC4246539

[CR75] Meng W, Chen Z, Jiang Q et al (2024) A multiplex real-time fluorescence-based quantitative PCR assay for calf diarrhea viruses. Front Microbiolo 14:1327291. 10.3389/fmicb.2023.1327291 10.3389/fmicb.2023.1327291 PMC10796610 38249490

[CR76] Mirhashemi M, Zintl A, Grant T et al (2015) Comparison of diagnostic techniques for the detection of Cryptosporidium oocysts in animal samples. Exp Parasitol 151:14–20. 10.1016/j.exppara.2015.01.018 25662435 10.1016/j.exppara.2015.01.018 PMC4406248

[CR77] Mohamed FF, Mansour SMG, El-Araby IE et al (2017) Molecular detection of enteric viruses from diarrheic calves in Egypt. Arch Virol 162:129–137. 10.1007/s00705-016-3088-0 27686074 10.1007/s00705-016-3088-0 PMC7086814

[CR78] Mooijman KA, Pielaat A, Kuijpers AFA (2019) Validation of EN ISO 6579-1 - Microbiology of the food chain - horizontal method for the detection, enumeration and serotyping of Salmonella - Part 1 detection of Salmonella spp. Int J Food Microbiol 2:288:3–12. 10.1016/j.ijfoodmicro.2018.03.022 10.1016/j.ijfoodmicro.2018.03.022 29803313

[CR79] Muktar Y, Mamo G, Tesfaye B, Belina D (2015) A review on major bacterial causes of calf diarrhea and its diagnostic method. J Vet Med Anim Health 7:173–185. 10.5897/JVMAH2014

[CR80] Nagamine K, Hase T, Notomi T (2002) Accelerated reaction by loop-mediated isothermal amplification using loop primers. Mol Cell Probes 16:223–229. 10.1006/mcpr.2002.0415 12144774 10.1006/mcpr.2002.0415

[CR81] Omoruyi BE, Nwodo UU, Udem CS, Okonkwo FO (2014) Comparative diagnostic techniques for Cryptosporidium infection. Molecules 19:2674–2683. 10.3390/molecules19022674 24566329 10.3390/molecules19022674 PMC6271508

[CR82] Pang B, Zhao C, Li L et al (2018) Development of a low-cost paper-based ELISA method for rapid Escherichia coli O157: H7 detection. Anal Biochem 542:58–62. 10.1016/j.ab.2017.11.010 10.1016/j.ab.2017.11.010 29158131

[CR83] Pang B, Yao S, Xu K et al (2019) A novel visual-mixed-dye for LAMP and its application in the detection of foodborne pathogens. Anal Biochem 574:1–6. 10.1016/j.ab.2019.03.002 30862446 10.1016/j.ab.2019.03.002

[CR84] Pansri P, Svensmark B, Liu G et al (2022) Evaluation of a novel multiplex qPCR method for rapid detection and quantification of pathogens associated with calf diarrhoea. J Appl Microbiol 133:2516–2527. 10.1111/jam.15722 35858716 10.1111/jam.15722 PMC9796748

[CR85] Papini R, Bonelli F, Montagnani M, Sgorbini M (2018) Evaluation of three commercial rapid kits to detect Cryptosporidium parvum in diarrhoeic calf stool. Ital J Anim Sci 17:1059–1064. 10.1080/1828051X.2018.1452055

[CR86] Peele D, Bradfield J, Pryor W, Vore S (1997) Comparison of identifications of human and animal source gram-negative bacteria by API 20E and crystal E/NF systems. J Clin Microbiol 35:213–216. 10.1128/jcm.35.1.213-216.1997 8968910 10.1128/jcm.35.1.213-216.1997 PMC229541

[CR87] Perumal V, Hashim U (2014) Advances in biosensors: principle, architecture and applications. J Appl Biomed 12:1–15. 10.1016/j.jab.2013.02.001

[CR88] Quan PL, Sauzade M, Brouzes E (2018) dPCR: a technology review. Sensors 18:1271. 10.3390/s18041271 29677144 10.3390/s18041271 PMC5948698

[CR89] Quinn PJ, Markey BK, Carter ME et al (2002) Veterinary microbiology and microbial disease. Textbook, Published by Blackwell, pp 113–116

[CR90] Ramos CP, Lopes EO, Oliveira Júnior CA et al (2020) Immunochromatographic test and ELISA for the detection of glutamate dehydrogenase (GDH) and A/B toxins as an alternative for the diagnosis of clostridioides (Clostridium) difficile-associated diarrhea in foals and neonatal piglets. Braz J Microbiol 51:1459–1462. 10.1007/s42770-020-00275-4 32363568 10.1007/s42770-020-00275-4 PMC7455628

[CR91] Razmi N, Hasanzadeh M, Willander M, Nur O (2020) Recent progress on the electrochemical biosensing of *Escherichia coli* O157:H7. Biosens (Basel) 10:54. 10.3390/bios10050054 10.3390/bios10050054 PMC7277213 32443629

[CR92] Saleh N, Allam T, Nayel M, Ahmed R (2022a) Molecular investigation of calf diarrhea in relation to changes in some immunological profiles. J Curr Vet Res 4:69–79. 10.21608/jcvr.2022.240872

[CR93] Saleh N, Allam T, Nayel M, Ahmed R et al (2022b) Hematological, serum biochemical and parasitological investigation of calf diarrhea. J Curr Vet Res 4:58–68. 10.21608/jcvr.2022.240868

[CR94] Santín M, James M, Ronald F (2007) Prevalence and molecular characterization of Cryptosporidium and Giardia species and genotypes in sheep in Maryland. Vet Parasitol 146:17–24. 10.1016/j.vetpar.2007.01.010 17335979 10.1016/j.vetpar.2007.01.010

[CR95] Sayar E, Keles I (2024) Investigation of the diagnostic and prognostic importance of tumor necrosis Factor-alfa (TNF-α), procalcitonin (PCT), interleukin-6 (IL-6) and haptoglobin (HP) in calves with neonatal diarrhea. Vet Immunol Immunopathol 277:110837. 10.1016/j.vetimm.2024.110837 39368395 10.1016/j.vetimm.2024.110837

[CR96] Schrader C, Schielke A, Ellerbroek L, Johne R (2012) PCR inhibitors- occurrence, properties and removal. J Appl Microbiol 113:1014–1026. 10.1111/j.1365-2672.2012.05384.x 22747964 10.1111/j.1365-2672.2012.05384.x

[CR97] Simpson KM, Callan RJ, Van Metre DC (2018) Clostridial abomasitis and enteritis in ruminants. Vet Clin North Am Food Anim Pract 34:155–184. 10.1016/j.cvfa.2017.10.010 29421028 10.1016/j.cvfa.2017.10.010 PMC7127689

[CR98] Smith G (2005) Fluid therapy in adult cattle. The north american veterinary conference – 2005 Proceedings

[CR99] Smith BP, Van Metre DC, Pusterla N (2020) Large animal internal medicine, 6th edn. ebook, Elsevier Publisher, pp 1810–1874

[CR100] Sobhy NM, Yousef SGA, Aboubakr HA et al (2020) Virulence factors and antibiograms of Escherichia coli isolated from diarrheic calves of Egyptian cattle and water buffaloes. PLoS ONE 15:e0232890. 10.1371/journal.pone.0232890 32392237 10.1371/journal.pone.0232890 PMC7213691

[CR101] Soltan MA, Tsai YL, Lee PA et al (2016) Comparison of electron microscopy, ELISA, real time RT-PCR and insulated isothermal RT-PCR for the detection of rotavirus group A (RVA) in feces of different animal species. J Virol Methods 235:99–104. 10.1016/j.jviromet.2016.05.006 27180038 10.1016/j.jviromet.2016.05.006 PMC7113751

[CR102] Su W, Du Y, Lian F et al (2022) Standards for collection, preservation, and transportation of fecal samples. Front Cell Infect Microbiol 12:783682. 10.3389/fcimb.2022.783682 35521221 10.3389/fcimb.2022.783682 PMC9065286

[CR103] Sultan K, Omar M, Desouky AY, El-Seify MA (2013) Molecular and phylogenetic study on Toxocara vitulorum from cattle in the mid-Delta of Egypt. J Parasit Dis 39:584–587. 10.1007/s12639-013-0373-7 26345077 10.1007/s12639-013-0373-7 PMC4554590

[CR104] Tabatabaei MS, Ahmed M (2022) Enzyme-Linked immunosorbent assay (ELISA). Methods Mol Biol 2508:115–134. 10.1007/978-1-0716-2376-3_10 35737237 10.1007/978-1-0716-2376-3_10

[CR105] Thiruppathiraja C, Saroja V, Kamatchiammal S et al (2011) Development of electrochemical based sandwich enzyme linked immunosensor for Cryptosporidium parvum detection in drinking water. J Environ Monit 13:2782–2787. 10.1039/c1em10372e 10.1039/c1em10372e 21897977

[CR106] Tothova C, Nagy O, Kovac G (2016) Serum proteins and their diagnostic utility in veterinary medicine: a review. Vet Med 61:475–496. 10.17221/19/2016VETMED

[CR107] Uslu NI, Ekic OD, Avci O (2023) ELISA–based point prevalence of enteropathogens in diarrheic calves in central Anatolia region of Turkey. Revista Científica 33:1–6. 10.52973/rcfcv-e33249

[CR108] Wang Q, Wang S, Beutin L et al (2010) Development of a DNA microarray for detection and serotyping of enterotoxigenic Escherichia coli. J Clin Microbiol 48:2066–2074. 10.1128/JCM.02014-09 20351209 10.1128/JCM.02014-09 PMC2884529

[CR109] Wang C, Xing K, Zhang G et al (2019) Novel ELISA based on fluorescent quenching of DNA-stabilized silver nanoclusters for detecting E. coli O157:H7. Food Chem 281:91–96. 10.1016/J.FOODCHEM.2018.12.079 30658771 10.1016/j.foodchem.2018.12.079

[CR110] Wang D, Gao H, Zhao L et al (2023) Detection of the dominant pathogens in diarrheal calves of Ningxia, China in 2021–2022. Front Vet Sci 10:1155061. 10.3389/fvets.2023.1155061 37138922 10.3389/fvets.2023.1155061 PMC10149748

[CR111] Wei X, Wang W, Dong Z et al (2021) Detection of infectious agents causing neonatal diarrhea on two large dairy farms in Yangxin County Shandong province, China. Front Vet Sci 7:589126. 10.3389/fvets.2020.589126 33614754 10.3389/fvets.2020.589126 PMC7892430

[CR112] Weigum SE, Castellanos-Gonzalez A, White AC, Richards-Kortum R (2013) Amplification-free detection of Cryptosporidium parvum nucleic acids with the use of DNA/RNA-directed gold nanoparticle assemblies. J Parasitol 99:923–926. 10.1645/12-132.1 23617738 10.1645/12-132.1 PMC3795944

[CR113] Woolums AR (2023) Serology in bovine infectious disease diagnosis. Vet Clin North Am Food Anim Pract 39:141–155. 10.1016/j.cvfa.2022.10.007 36731994 10.1016/j.cvfa.2022.10.007

[CR114] Younis EE, Ahmed AM, El-Khodery SA et al (2009) Molecular screening and risk factors of enterotoxigenic Escherichia coli and Salmonella spp. in diarrheic neonatal calves in Egypt. Res Vet Sci 87:373–379. 10.1016/j.rvsc.2009.04.006 10.1016/j.rvsc.2009.04.006 PMC7111881 19419742

